# Subjective Well-Being, Test Anxiety, Academic Achievement: Testing for Reciprocal Effects

**DOI:** 10.3389/fpsyg.2015.01994

**Published:** 2016-01-08

**Authors:** Ricarda Steinmayr, Julia Crede, Nele McElvany, Linda Wirthwein

**Affiliations:** ^1^Department of Psychology, Technical University of DortmundDortmund, Germany; ^2^Institute for School Development Research (IFS), Technical University of DortmundDortmund, Germany

**Keywords:** subjective well-being, academic achievement, test anxiety, achievement emotions, adolescence

## Abstract

In the context of adolescents’ subjective well-being (SWB), research has recently focused on a number of different school variables. The direction of the relationships between adolescents’ SWB, academic achievement, and test anxiety is, however, still open although reciprocal causation has been hypothesized. The present study set out to investigate to what extent SWB, academic achievement, and test anxiety influence each other over time. A sample of *N* = 290 11th grade students (*n* = 138 female; age: *M* = 16.54 years, *SD* = 0.57) completed measures of SWB and test anxiety in the time span of 1 year. Grade point average (GPA) indicated students’ academic achievement. We analyzed the reciprocal relations using cross-lagged structural equation modeling. The model fit was satisfactory for all computed models. Results indicated that the worry component of test anxiety negatively and GPA positively predicted changes in the cognitive component of SWB (life satisfaction). Worry also negatively predicted changes in the affective component of SWB. Moreover, worry negatively predicted changes in students’ GPA. Directions for future research and the differential predictive influences of academic achievement and test anxiety on adolescents’ SWB are discussed with regard to potential underlying processes.

## Introduction

Both academic achievement and subjective well-being (SWB) play a major role in people’s lives. Academic achievement is important as it strongly shapes a person’s life chances (Steinmayr et al., 2014) and SWB impacts on important variables such as basic psychological needs (Tian et al., 2014) and social connections (Shankar et al., 2015). Knowing what influences these variables is of utmost importance as this is the basis for effective interventions (Byrnes and Miller, 2007).

A lot of research has investigated motivational and cognitive variables as predictors of school achievement (e.g., Steinmayr and Spinath, 2009). Among emotional variables, test anxiety has been the construct most frequently investigated (e.g., Schwarzer, 1990; Seipp, 1991). Furthermore, studies concentrated on the association between mental illnesses, e.g., depression, and school achievement (e.g., Chong et al., 2009). However, over the last few decades, there has been a growing interest in research on students’ perceptions of their well-being as an indicator of mental health (Bradshaw et al., 2007; Kosher et al., 2014). While many studies have already examined individual determinants of students’ SWB such as personality variables (e.g., Singh and Lal, 2012; Anglim and Grant, 2014), research focusing on school variables is still scarce. Some authors already pointed to the importance of academic achievement for adolescents’ SWB (e.g., Adelman and Taylor, 2006) and indeed, a few studies demonstrated a cross-sectional association between both variables (Kirkcaldy et al., 2004; Crede et al., 2015; see also Suldo et al., 2008). However, the direction of this relationship is still unknown as there are no longitudinal studies investigating the two variables. As there are strong theoretical arguments supporting a reciprocal relationship (see Mortimore, 1991; Samdal et al., 1999), we aimed at examining the direction of the SWB-academic achievement relationship in the present study to fill this research gap.

Although test anxiety is considered as one aspect of emotional well-being (Rüppel et al., 2015), the relationship between test anxiety and students’ SWB has not been the focus of many studies yet. However, there are theoretical reasons to believe that these constructs are interrelated. Furthermore, recent studies demonstrated stable associations between test anxiety and other variables indicating an emotional equilibrium (Pekrun et al., 2002; Ringeisen and Buchwald, 2010). Last but not least, as test anxiety is considered as one aspect of emotional well-being and several studies demonstrated a link between text anxiety and academic achievement (e.g., Steinmayr et al., 2014), it is important to additionally consider test anxiety in a study exploring the link between SWB and academic achievement longitudinally. Thus, the present study set out to examine whether change in SWB is predicted by academic achievement and text anxiety and vice versa. Results regarding the direction of these relationships might be useful for developing education interventions to enhance children’s SWB in the context of school (e.g., Bird and Markle, 2012). Moreover, the present study is set up with a special focus on adolescence because this can be seen as a phase characterized as period of heightened stress (Spear, 2000) due to many changes experienced such as a desire of independence, challenges of social and peer interactions and school stress management (e.g., Blakemore, 2008).

### SWB and Academic Achievement

Subjective well-being describes individuals’ cognitive evaluations of their lives as a whole (i.e., global life satisfaction) as well as reports on affective well-being (see Diener et al., 1999). Affective experiences include adolescents’ reports of pleasant emotions (e.g., joy, excitement), and negative emotions (e.g., sadness, anger; Bradshaw et al., 2011). Life satisfaction, the cognitive component of SWB, is known as the most stable component of SWB (e.g., Suldo et al., 2006).

Among the indicators of adolescents’ well-being, school variables have been investigated more closely only recently. Here, the association between academic achievement and life satisfaction (cf. Crede et al., 2015) is important. For example, Gilman and Huebner (2006) found global life satisfaction to be positively related to adolescents’ grade point averages (GPAs) and attitude toward school and negatively correlated with psychopathological problems such as depression or social stress. Similarly, Proctor et al. (2010) demonstrated that adolescents’ life satisfaction scores were significantly positively related to their GPAs. The correlations between life satisfaction and academic achievement ranged from *r* = 0.12 (Verkuyten and Thijs, 2002) to *r* = 0.32 (Gilman and Huebner, 2006). The association between affective well-being and academic achievement is more heterogeneous: while it has produced at least small positive associations in some studies (e.g., Ying and Liese, 1991), no significant correlation between positive affect as part of SWB and students’ GPA was found in studies with university students (Liao and Wei, 2014).

To date, especially fundamental longitudinal studies on the relationship between SWB and academic achievement illuminating the causal ordering of the two constructs are still lacking. On the one hand, some results highlighted the higher academic functioning of adolescents with high SWB scores and low levels of psychopathology. For example, Suldo and Shaffer (2008) assessed SWB (i.e., life satisfaction, positive affect, and negative affect) and internalizing psychopathology, as well as measures of physical health, social functioning, and attitudes toward school. Results demonstrated that academically successful students showed good mental health whereas adolescents in the “vulnerable” group had worse results in a standardized measure of reading achievement (see also Greenspoon and Saklofske, 2001). However, even though this study might be a first hint that academic achievement might impact on SWB it does not allow for a definite conclusion because of methodological reasons. First, this study did not control for academic achievement at first measurement occasion and second, it did not assess a direct impact of academic achievement on adolescents’ SWB for which further longitudinal investigation is necessary.

On the other hand, there is also theoretical support for the impact of children’s SWB on their school performance. Well-adapted students or students with high SWB may be more academically successful and a ‘happy’ student may eventually demonstrate higher levels of SWB resulting in higher academic functioning, which is supported by findings of major experimental studies in emotion research. For example, Meinhardt and Pekrun (2003) found that emotional states induced by affective pictures or recollection of critical life events reduced the cognitive resources available for task purposes. These results may be first experimental hints that the emotional component of SWB predicts changes in students’ academic achievement.

In sum, one can argue that academic achievement predicts changes in SWB and that both components of SWB may predict changes in students’ academic achievement. This can also be underlined theoretically because high achievement scores may also increase students’ SWB, which eventually motivates students to get better grades, leading to a phenomenon which is called ‘the good circle’ in education (Samdal et al., 1999). Consequently, one can assume that academic achievement predicts changes in students’ SWB and that SWB predicts changes in academic achievement.

### Test Anxiety and SWB

Test anxiety affects students in the field of assessment and evaluation of their abilities and achievements. According to Spielberger (1972) test-anxious persons are characterized by acquired habits and attitudes that involve negative self-perceptions and expectations. These self-deprecating attitudes dispose test-anxious persons to experience fear and heightened physiological activity in situations such as examinations in which they interpret and respond to events in the environment.

Today most authors view test anxiety as being composed of two dimensions known as emotionality, an affective-physiological dimension, and worry, a cognitive dimension (Liebert and Morris, 1967; Cassady and Johnson, 2002). Worry typically involves negative thoughts, self-criticism or concerns about the negative consequences of failure that occur during testing situations (Zeidner, 1998). Emotionality, on the other hand, describes a person’s appraisal of his or her physiological state such as tension, tight muscles, accelerated heart rate or nervousness (Zeidner, 1998, 2007).

Test anxiety can affect adolescents in every field of life (Lufi et al., 2004). Detailed research on the relationship between SWB and test anxiety is still scarce. However, a number of studies assessing test anxiety in the context of emotions and emotional states (for a review see Zeidner, 1998) and forms of mental illness (e.g., Liu and Lu, 2012; Akinsola and Nwajei, 2013) found associations between the constructs. For example, in a sample of senior secondary school students, Akinsola and Nwajei (2013) demonstrated that test anxiety and depression were positively related (*r* = 0.32). As test-anxiety is considered as one aspect of a person’s emotional well-being, describing the opposite mental state to mental illness (Rüppel et al., 2015), a negative link between SWB and test anxiety is likely. However, we are not aware of any cross-sectional or longitudinal study that examined test anxiety in the context of SWB so far.

Concerning the causal ordering of the test-anxiety-SWB relationship, there are reasons to hypothesize that test anxiety predicts changes in students’ SWB. This is supported by the transactional model of test-related emotions which has been applied to the study of test anxiety (Spielberger and Vagg, 1995; Zeidner, 1998; Ringeisen and Buchwald, 2010). The transactional model stems from cognitive-motivational research and postulates that test anxiety is not only associated with appraisals of threat, but also with other distinct negative and positive evaluations, and accompanying emotions (Ringeisen and Buchwald, 2010). Thus, test anxiety may predict changes in the emotional and cognitive components of SWB. Moreover, test anxiety has been found to relate positively to higher emotion-focus (e.g., trying to control anxiety symptoms) and greater avoidance (e.g., trying not to think of the test; Zeidner, 2007). Therefore, one can hypothesize that both components of test anxiety negatively affect changes in both components of SWB.

However, it is also of interest to find out whether the components of SWB predict changes in test anxiety. Especially the cognitive component of SWB (life satisfaction) may contribute to the development of test anxiety. This assumption is underlined by the Self-referent executive function (S-REF) theory of emotional distress (Zeidner and Matthews, 2005). The theory builds on earlier work on transactional stress processes to specify how executive processing of self-referent information leads to anxiety. It assumes that this processing is shaped by declarative and procedural self-knowledge held in a person’s long-term memory. Self-referent processing is generated by threatening cognitions caused by external stimuli. In the short-term, worry is generated because a person accesses negative self-beliefs, for example lack of academic competence, and chooses counterproductive coping strategies. Eventually, these mechanisms may lead to test anxiety. In the longer term, distress may be maintained by dysfunctional styles of person-situation interaction (Zeidner and Matthews, 2005). Consequently, a well-adjusted person with higher life satisfaction scores may be able to modify self-knowledge and learn of more effective coping strategies, such as studying harder after a poor performance. A person scoring low on the cognitive component of SWB, however, may strengthen and elaborate negative self-beliefs, such as being unable to cope with examinations. Thus, a student with negative life satisfaction scores may have greater risks of becoming a test-anxious student due to a greater attention focus on the feared event of an examination and negative coping strategies.

Summing up, we hypothesized that the relations between test anxiety and SWB may be reciprocal. However, both directions are inferred from theories only indirectly dealing with test anxiety or SWB. Thus, we exploratively investigate the causal ordering between text anxiety and SWB in the present study.

### Test Anxiety and Academic Achievement

Although there are several studies examining the association between test anxiety and academic achievement, longitudinal research focusing on the reciprocal effects between both variables is scarce (Seel, 2012). Most cross-sectional studies demonstrated negative correlations between test anxiety and academic achievement (e.g., Cassady and Johnson, 2002; Smith and Smith, 2002; Nicholson, 2009). For example, in a meta-analysis conducted by Schwarzer (1990) and Seipp (1991), a negative correlation of *r* = -0.21 was found between worry and academic achievement: Students with high levels of worry are expected to show lower academic achievement scores. Opposed to worry, emotionality has been found to have only little impact on students’ academic achievement (Deffenbacher, 1980). Whereas Wigfield and Meece (1988) found that emotionality showed higher negative associations with performance than worry, Meece et al. (1990) examined the effects of math anxiety on students’ grades longitudinally but found no significant direct effects. Concerning standardized achievement scores, however, emotionality was negatively related to achievement test scores including language achievement (*r* = -0.10), math (*r* = -0.03), social studies (*r* = -0.07), and science (*r* = -0.04; O’Neil and Fukumura, 1992).

Test anxiety may have a negative impact on academic achievement, which is emphasized by Eysenck’s Attentional Control Theory (Eysenck et al., 2007). According to this theory, anxiety impairs efficient functioning of students’ attentional system and increases the extent to which processing efficiency depend on attentional control. Thus, it explains the effects of anxiety on attentional processes and on students’ cognitive performance. In this respect, anxiety may reduce a students’ attentional focus to the examination task and instead makes a student focus his or her attention to other stimuli such as thoughts of worry or other distracting aspects which are not relevant to the task. Thus, we assume that test anxiety, especially the worry component, decreases school performance.

With regard to the effects of students’ academic achievement on test anxiety, only a small number of studies exist so far. Some authors hypothesize that the experience of academic success or failure increases negative emotions concerning situations in which academic performance is measured (Eccles and Wigfield, 2002). In this vein, Galassi et al. (1981) demonstrated that low academic achievement was likely to increase students’ test anxiety about upcoming exams and that students with high GPAs reported more bodily sensations indicative of arousal than students with low GPAs.

Summing up, the correlations between test anxiety and academic achievement may be caused by reciprocal causation (see also Seel, 2012). This is underlined by longitudinal evidence available in this field suggesting that test anxiety and students’ learning outcomes are in fact linked by reciprocal causation across school years (Meece et al., 1990; Pekrun, 1992). However, we are not aware of any study that investigated the associations between text anxiety and grades over time. Furthermore, the additional investigation of text anxiety in the context of academic performance and SWB is important as test anxiety is considered as an emotional (negative) component of SWB which is related to academic performance. Thus, only when controlling for test anxiety, one can evaluate the actual relationship between SWB and academic performance.

### The Present Study

As outlined, reciprocal relations between students’ SWB and academic achievement, as well as between SWB and test anxiety have not been investigated so far. It is one aim of the study to exploratively test for the reciprocal relationships between those constructs. Knowing which construct impacts on another over-time is a mandatory precondition for planning any interventions (Byrnes and Miller, 2007). Based on theories and prior research we formed some hypotheses.

Cross-sectional studies investigating the associations between SWB and academic achievement found small positive correlations (e.g., Gilman and Huebner, 2006). Longitudinal research is lacking but there are hints that students with initial high SWB scores show higher academic functioning (e.g., Greenspoon and Saklofske, 2001). Hence, one can hypothesize that high academic achievement has positive effects on SWB. But there are also reasons to assume that SWB may positively predict changes in academic achievement (e.g., Suldo et al., 2011). For the present study, we hypothesized that students’ achievement positively predicts changes in the cognitive and emotional components of SWB. Moreover, we hypothesized that both components of SWB positively predict changes in students’ achievement.

The longitudinal relationship between SWB and test anxiety has not been examined yet. Due to the lack of research we focused on studies investigating the relationship between test anxiety and emotions or depression (e.g., Zeidner, 1998). Here, positive associations between negative emotions and test anxiety were found. Hence, we assumed a negative relationship between the components of SWB and test anxiety. Drawing on theories regarding the direction of the relationship, the two components of test anxiety (worry, emotionality) might negatively predict changes in both components of SWB (e.g., Ringeisen and Buchwald, 2010). However, one can also expect that SWB negatively predicts changes in both components of test anxiety. According to Zeidner and Matthews (2005), especially the cognitive component of SWB might have effects on test anxiety. A person scoring high on life satisfaction might develop lower levels of test anxiety due to coping styles or positive self-beliefs that are accompanied with a high life satisfaction. In sum, there are hints that SWB and test anxiety might be reciprocally related and that both components of test anxiety might have negative influences on the changes in both components of SWB. In addition, life satisfaction and mood level might negatively predict changes in students’ test anxiety.

Finally, we were interested in the relationship between test anxiety and academic achievement. Based on previous findings, especially worry might negatively predict changes in academic achievement (e.g., Seipp, 1991; Eysenck et al., 2007). However, according to Galassi et al. (1981), students’ GPA might also predict changes in worry and emotionality, which is why we hypothesized students’ GPA and test anxiety to be reciprocally related.

Beside the reciprocal relationships between construct dyads we were also interested in how all constructs would interact together as all focused constructs were interrelated. Furthermore, gender differences are known for some of the investigated constructs. For example, the proportion of students who report themselves as highly test anxious is known to differ by gender with the proportion being significantly higher in girls (concerning both worry and emotionality) with larger differences found for the emotionality component of test anxiety (see Zeidner and Schleyer, 1999; Steinmayr and Spinath, 2014). Thus, we additionally controlled for gender.

## Materials and Methods

### Sample and Procedure

In the present study, *N* = 290 German high school students (*n* = 138 female) participated. The first measurement occasion took place in fall of 2008 and the second one about 1 year later in fall of 2009. At the first measurement occasion students were in 11th grade and at the second one, they attended 12th grade (about 1 year away from graduating from this type of school). The school attended by the students represents the highest academic track in the German school system (“Gymnasium”). In the German education system, these final years of secondary education are highly relevant for students as they prepare for Abitur examinations, which allow them to study at university. Furthermore, this period is accompanied by further changes. At the beginning of the first measurement occasion, students were no longer taught in one class in all subjects but faced the challenge to get used to a new course system. This included more freedom of choice in school subjects despite some restrictions as they were not allowed to drop main subjects such as German or math. In the course of this year, however, the relevance of students’ school achievement increased as their GPAs became more and more important for the GPA of their school leaving certificate. As the GPA of the school leaving certificate of this school track is often the only criterion for university application, academic achievement in this school phase is highly important for students’ following career options and life opportunities. The mean age in our sample was *M* = 16.53 years (*SD* = 0.57) at the first measurement occasion. Testing at both measurement occasions took place during regular school day, and participation was optional. Before testing we received written consent from all parents of the under-aged students. At both measurement occasions, the scales of interest for the present study were given to the students as a part of a larger testing battery. The students were tested by trained research assistants and psychology university students.

### Measures

#### SWB

Subjective well-being was measured using the Habitual SWB Scale (HSWBS; Dalbert, 2003). It consists of a mood-level scale (Dalbert, 1992), which consists of six items assessing emotional components of SWB, and a satisfaction with life scale (Dalbert et al., 1984), which consists of seven items assessing cognitive components of SWB. The items of this life satisfaction scale are comparable with those of the life satisfaction scale developed by Diener et al. (1985) and assess how satisfied one is with his or her present life (three items; e.g., “I am satisfied with my life”), his or her life in the past (two items; e.g., “When I look back on my life so far, I am satisfied”) and what he or she expects for his or her life in the future (two items; e.g., “I believe that much of what I hope for will be fulfilled”). The mood level scale is a German short version of the Mood Level Scale by Underwood and Froming (1980). It assesses the absence of negative emotions and whether someone mostly experiences positive emotions (e.g., “Mostly I am happy”). In the present study we only used five of the six items of the mood-level scale because the phrasing of the sixth item is not commonly used anymore and might have irritated the participants (“Mostly I feel like I am brimming with joy”). However, the internal consistencies of both scales are comparable with those found by Dalbert (2003): mood-level scale: α = 0.82 compared to α_t1_ = 0.86 and α_t2_ = 0.85 in the present sample; life satisfaction scale α = 0.88 compared to α_t1_ = 0.88 and α_t2_ = 0.93 in the present sample.

#### Test Anxiety

Test anxiety was measured using a short version (Schwarzer and Jerusalem, 1999) of the German Test Anxiety Inventory (TAI-G; Hodapp, 1991, 1995), a revised multidimensional version of Spielberger’s Test Anxiety Inventory (TAI; Spielberger et al., 1981). This short version consists of ten items with two scales: Worry (five items) and emotionality (five items). The participants were asked to answer on a four-point Likert rating scale ranging from (1) “almost never” to (4) “almost always” regarding their feelings and general thoughts in test situations. The scale worry assesses how much one worries in a test situation (e.g., “I ask myself whether my performance will be sufficient”). The scale emotionality assesses the physiological and excitement-related components in a test situation (e.g., “My heart is in my mouth”). Cronbach’s α coefficients were high for both scales and measurement points (emotionality: α_t1_ = 0.86 and α_t2_ = 0.86; worry: α_t1_ = 0.80 and α_t2_ = 0.84).

#### Academic Achievement

At both measurement occasions academic achievement was measured by GPA as indicated by students’ last and subsequent report cards. The school delivered report cards for all students. In Germany, grades are coded so that “1” indicates outstanding achievement and “6” indicates the poorest achievement. Grades were reversed to facilitate interpretation of the results so that higher scores indicated better performance. The subjects German and math were mandatory and thus all students had grades in these subjects. With regard to foreign language, science, and social science, students were allowed to choose courses. Grades of the different courses within each category were summed up as indicators of academic achievement in these domains.

### Statistical Analyses

#### Missing Data

The main reason for students missing class at the day testing took place was illness. We are not aware of any other reasons for children to miss testing day that might be systematically related to the testing and investigated variables. At each measurement occasion, approximately 30 students did not participate. 229 students participated at both measurement occasions. We tried to explore the nature of the sample depletion by comparing children with missing measurements with the sample of complete data sets. We found no systematic differences in any investigated variable, age, or sex. Furthermore, there were only small amounts of missing data for individual items (less than 1%). As proposed by several authors we accounted for missing data by means of applying the full information maximum likelihood (FIML) estimations (Enders and Bandalos, 2001; Newman, 2003).

#### Measurement Models and Measurement Invariance

First, the measurement model for each construct was tested based on the items of measurement occasion 1 using Amos 22. Second, we ensured that the construct validity of the focused constructs did not change over time (cf. Meredith, 1993). To this end, we tested the longitudinal measurement invariance of the different constructs. Each model included two correlated latent constructs that represented the construct of interest at measurement occasions 1 and 2. Within the scope of this analysis, we compared a more complex model without equality constraints over time with a more parsimonious model wherein factor loadings of the manifest variables are constraint to be equal over time. Besides the frequently used chi-square (χ^2^) difference test, we employed the following guidelines to compare the complex with the more parsimonious model: If the differences between the Comparative Fit Index (CFI) of the parsimonious model and the CFI of the configural model is <0.010 (Cheung and Rensvold, 2002) and the difference between the Root Mean Square Error of Approximation (RMSEAs) of the two models is <0.015 (Chen, 2007), there is no substantial decrease in model fit and it provides support for the more parsimonious model, i.e., that the construct validity of the focused constructs did not change over time.

#### Cross-Lagged Models

We aimed at testing the reciprocal effects between different components of SWB (life satisfaction and mood-level), text anxiety (emotionality and worry), and academic achievement. We followed the guidelines for analyses of reciprocal effects between concepts in longitudinal designs provided by Marsh (1990) and Marsh et al. (1999). Data were also analyzed by computing longitudinal SEM with Amos 22.

For each hypothesis, we set up one cross-lagged model. Spanning the time of 1 year, the models employed two measurement occasions. The two latent constructs in each model at each measurement occasion were indicated by the items of the corresponding scale. To control for potential memory effects and positively overestimated stabilities between the different measurement occasions, models were set up with correlated uniqueness between the same items which were collected at subsequent measurement occasions.

For the evaluation of the overall model fit, three different fit indices were used (see Hu and Bentler, 1999): *X*^2^-value, RMSEA, and CFI. The *X*^2^-value should be relativized with regard to the model’s degrees of freedom (*X*^2^/df) and this term should be smaller than 3 (Carmines and McIver, 1981; Marsh and Hocevar, 1985). A RMSEA < 0.05 indicates a very good model fit and a RMSEA < 0.09 is still an indicator of a reasonable error of approximation in smaller samples (e.g., *N* < 300; Browne and Cudeck, 1993). According to Hu and Bentler (1999), it is difficult to provide a recommended range for the CFI because in some cases even a CFI < 0.90 can indicate a reasonable model fit, which is why one usually looks for a CFI < 0.95. However, in small sample sizes (*N* < 300) even a CFI ≥ 0.90 can indicate an acceptable fit (Bentler, 1990; Hu and Bentler, 1995). Thus, the cut-off scores in the present sample are *X*^2^/df < 3; RMSEA < 0.09 and a CFI > 0.90.

## Results

### Descriptive Statistics

Means, standard deviations, internal consistencies of and intercorrelations between all measures are depicted in **Table 1.**

**Table 1:** Means (M), standard deviations (SD), internal consistencies (α), and intercorrelations among all predictors.

	Descriptives			Intercorrelations									
		
	*M*	*SD*	α	(1)	(2)	(3)	(4)	(5)	(6)	(7)	(8)	(9)	(10)
Measurement occasion 1													
(1) Life satisfaction	5.19	1.03	0.88		0.68	0.004	-0.23	0.10	0.61	0.50	-0.06	-0.19	0.11
(2) Mood	5.06	1.22	0.86			-0.03	-0.13	0.02	0.50	0.63	-0.08	-0.14	0.03
(3) Emotionality	1.98	0.66	0.81				0.62	-0.02	-0.06	-0.08	0.60	0.49	-0.09
(4) Worry	2.66	0.70	0.86					-0.11	-0.21	-0.24	0.49	0.64	-0.21
(5) GPA	2.73	0.57	0.78						0.19	0.01	-0.02	-0.15	0.73
Measurement occasion 2													
(6) Life satisfaction	5.23	1.19	0.92							0.71	-0.15	-0.14	0.18
(7) Mood	5.21	1.16	0.85								-0.12	-0.17	0.02
(8) Emotionality	1.88	0.65	0.84									0.69	-0.06
(9) Worry	2.46	0.75	0.85										-0.21
(10) GPA	2.85	0.59	0.82										


**N* = 290; The SWB scales (life satisfaction and mood) ranged from 1 to 7, the test anxiety scales (Emotionality and Worry) from 1 to 4 with 1 indicating lower values. *r* ≥∣0.13∣, *p* < 0.05; *r* ≥∣0.18∣, *p* < 0.01.*

Subscales of test anxiety as well as those of SWB were highly intercorrelated. Both life satisfaction and mood negatively correlated with worry at both measurement occasions. Academic achievement was positively associated with life satisfaction and negatively associated with worry at both measurement occasions.

### Measurement Models and Measurement Invariance

The measurement models were set up based on the theoretical foundation of the scales. Academic achievement was indicated by five manifest variables: the grade in German, the grade in math, the average grade in science subjects, the average grade in social sciences classes and the average grade in foreign language classes. As these subjects represent different domains, the correlated errors were allowed for math and science, for native language and foreign language as well as for native language and social sciences. After these adjustments were made, the model fit was excellent: *X*^2^ (*df* = 2) = 0.19, *p* = 0.82; *CFI* = 1.00; *RMSEA* < 0.001. The worry scale of the TAI-GE had a satisfactory model fit after correlating the two measurement errors of the two items assessing lack of confidence in one’s competencies *X*^2^ (*df* = 4) = 2.29, *p* = 0.06; *CFI* = 0.99; *RMSEA* = 0.067. Apparently, this aspect is independent from one’s general worries concerning an exam. The emotionality scale of the TAI-GE had an excellent model fit after correlating the measurement errors of those items assessing physiological signs of excitement and those items that measure the emotional components of excitement (*X*^2^ (*df* = 4) = 1.95, *p* = 0.14; *CFI* = 0.996; *RMSEA* = 0.057).

Concerning the satisfaction with life scale we correlated the measurement errors of those items each assessing satisfaction with one’s present life, past life and future perspectives. The model fit of the satisfaction of life scale was satisfactory: *X^2^* (*df* = 9) = 2.22, *p* = 0.02; *CFI* = 0.99; *RMSEA* = 0.065. The same was true for the mood scale for which no model adjustment seemed theoretically sensible and empirically necessary: *X*^2^ (*df* = 5) = 1.91, *p* = 0.09; *CFI* = 0.99; *RMSEA* = 0.056.

Concerning the measurement invariance over the two measurement occasions, we found all scales to be measurement invariant over time according to the criteria set by Cheung and Rensvold (2002) and Chen (2007). None of the measurement invariance restrictions indicated a substantial change in model fit: life satisfaction (Δ *CFI* = 0.005; Δ *RMSEA* = 0.004), mood (Δ *CFI* = 0.008; Δ *RMSEA* < 0.001), emotionality (Δ *CFI* = 0.002; Δ *RMSEA* = 0.008), worry (Δ *CFI* = 0.001; Δ *RMSEA* = 0.005), GPA (Δ *CFI* = 0.002; Δ *RMSEA* = 0.012).

### Cross-Lagged Models

As we found positive correlations among most constructs, we first did not check all constructs simultaneously as multicollinearity might distort the result pattern. For example, it would be possible that both worry and emotionality significantly predict changes in grades from measurement point 1 to measurement point 2 if they were considered separately. However, due to the fact that these constructs are correlated, it might be that neither construct was a significant predictor of grades if both constructs were considered simultaneously. This might be the fact as the path coefficient in the latter model indicates the unique effect of each construct on the criterion, which might not be significant opposed to the total effects worry and emotionality have in separate models (cf. Marsh et al., 2004). In order to test the effect of each construct on the change in another, we first set up causal ordering models for the various pairs of constructs testing for bivariate reciprocal effects. We were not interested in the reciprocal effects between two subcomponents of one construct (e.g., worry and emotionality). Thus, we tested eight cross-lagged models incorporating two constructs each. The results are depicted in **Table 2.**

**Table 2:** Model Fit (*X*^2^, CFI, RMSEA) of the Cross-lagged Models as well as Path Coefficients Relating Time 1 Constructs (Worry, Emotionality, Mood-level, Satisfaction with Life, Academic Achievement) to the Corresponding Time 2 Constructs.

Model	Tested construct	*X^2^/df*	CFI	RMSEA	Stability path 1	Stability path 2	Construct 1) →Construct 2)	Construct 2) →Construct 1)
(1)	1) Emotionality ↔	1.48	0.971	0.041	0.656^∗∗∗^	0.664^∗∗∗^	-0.064	-0.088
	2) Life satisfaction							
(2)	1) Worry ↔	1.38	0.976	0.036	0.756^∗∗∗^	0.631^∗∗∗^	-0.125^∗^	-0.019
	2) Life satisfaction							
(3)	1) GPA ↔	1.89	0.946	0.056	0.830^∗∗∗^	0.650^∗∗∗^	0.142^∗^	0.036
	2) Life satisfaction							
(4)	1) Emotionality ↔	1.73	0.955	0.050	0.635^∗∗∗^	0.676^∗∗∗^	-0.086	-0.075
	2) Mood Level							
(5)	1) Worry ↔	1.62	0.960	0.046	0.757^∗∗∗^	0.624^∗∗∗^	-0.225^∗∗∗^	-0.010
	2) Mood Level							
(6)	1) GPA ↔	2.44	0.912	0.071	0.833^∗∗∗^	0.678^∗∗∗^	0.039	0.031
	2) Mood Level							
(7)	1) Emotionality ↔	2.34	0.922	0.068	0.657^∗∗∗^	0.823^∗∗∗^	-0.045	0.003
	2) GPA							
(8)	1) Worry ↔	1.91	0.942	0.056	0.740^∗∗∗^	0.807^∗∗∗^	-0.104^∗^	-0.085
	2) GPA							


*Construct 1 is the first mentioned construct and construct 2 the last mentioned construct in the specific model. GPA, Grade Point Average. ^∗^*p* < 0.05, ^∗∗∗^*p* < 0.001.*

Model fit indices of the different models indicated a satisfactory fit to the data at least (0.91 ≤ CFI ≤ 0.98, 0.04 ≤ RMSEA ≤ 0.07). All constructs had a high stability concerning the time span of 1 year ranging from 0.63 (life satisfaction) to 0.82 (GPA). Emotionality assessed at the first measurement occasion did not predict changes in life satisfaction and vice versa (Model 1). A decrease in life satisfaction was predicted by worry at measurement occasion 1 but life satisfaction at measurement point 1 did not predict the change in worry from measurement point 1 to 2 (Model 2). An increase in life satisfaction was predicted by GPA at measurement occasion 1 but the reciprocal effect could not be found (Model 3). Change in the mood level scale was only significantly predicted by worry at measurement point 1. The more students worried at measurement occasion 1 the more negatively changed their mood level from measurement occasion 1 to measurement occasion 2 but mood level at measurement point 1 did not predict change in worry (Model 5). No reciprocal effects were found between emotionality and mood level (Model 4) and between GPA and mood level (Model 6). Emotionality at measurement point 1 did not predict change in GPA and GPA at measurement point 1 did not predict change in Emotionality (Model 7). But change in academic achievement was only predicted by worry at measurement point 1. The more students worried at measurement occasion 1 the more negatively changed their GPA from measurement occasion 1 to measurement occasion 2, but GPA at measurement point 1 did not predict change in worry (Model 8).

**FIGURE 1:**
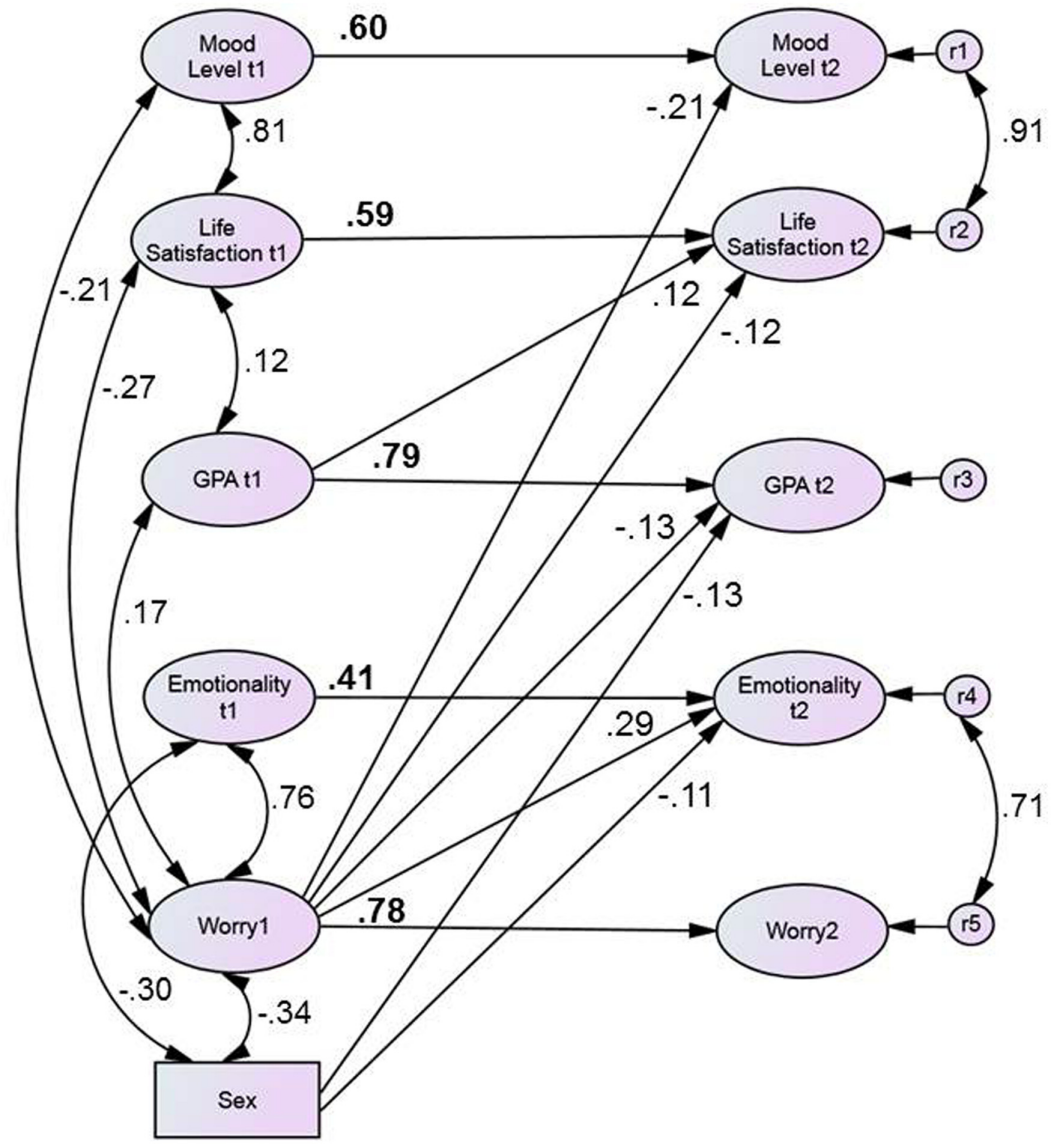
**SEM incorporating all constructs controlling for each other.** Only significant paths and correlations (*p* < 0.05) were considered. Numbers in bold represent stability paths.

Summing up, worry at measurement point predicted change in GPA and both components of SWB (life satisfaction and mood level). Only GPA at measurement point 1 was another significant predictor of life satisfaction. In order to test whether these effects still hold if all constructs are tested simultaneously and if we control for gender we set up a model including all constructs at both measurement occasions. All constructs at measurement point 1 were correlated with each other. Thus, the cross-lagged path weights from one construct at measurement point 1 to another construct at measurement point 2 indicates the unique effect of this construct on the other controlling for all other constructs at measurement point 1. Only significant paths were considered and are depicted in **Figure 1.** Model fit of this model was satisfactory (*X*^2^/*df* = 1.59; *CFI* = 0.91; *RMSEA* = 0.045).

**Figure 1** demonstrates that worry at measurement point 1 still significantly predicted change in GPA and in both components of SWB (life satisfaction and mood level). Furthermore, worry at measurement point 1 predicted change in emotionality. Gender significantly predicted change in GPA as well as in emotionality. For female students, grades changed for the better and emotionality increased. GPA at measurement point 1 was still another significant predictor of life satisfaction. Compared to the bivariate cross-lagged models, cross-lagged paths were slightly diminished due to multi-collinearity.

## Discussion

The aim of the present paper was to explore the reciprocal effects between SWB, academic achievement, and test anxiety in a sample of 290 German high school students. The results contribute to an ongoing debate on improving adolescents’ SWB in the context of education because students, especially on the academic track, face high demands at school today and become more aware of individual differences in ability and achievement during adolescence as a phase of development (Wigfield et al., 2006). To our knowledge, no single longitudinal study investigating the relationship between these variables in middle or late adolescence has been carried out before.

First, we looked at reciprocal relations between SWB and students’ GPA. We hypothesized that students’ GPA positively predicts changes in the cognitive and emotional components of SWB. However, students’ GPA only predicted changes in the cognitive component (life satisfaction), but not in the affective component of SWB (mood-level). It is already known that school success may predict many long-term positive outcomes, such as higher education studies, better job possibilities, or positive self-perceptions (e.g., Salmela-Aro and Upadyaya, 2012; Wang and Peck, 2013). Greenspoon and Saklofske (2001) already found hints that students with better school outcomes were more satisfied than students with less favorable school engagement trajectories. In the present study, we demonstrated that students’ academic achievement directly predicts changes in the cognitive component of well-being. On the one hand, this effect can be explained by the finding that life satisfaction is the most stable and key indicator of children’s SWB (Suldo et al., 2006). On the other hand, students’ mood-level (positive and negative affect) may be rather influenced more strongly by diverse activities that are not related to school environment, such as family and leisure events. For example, in study with 14-year old adolescents with depressed mood, adolescents participating in after-school activities (e.g., sports teams, music clubs, community service) reported lower levels of depressed mood compared to adolescents not participating in such activities (Mahoney et al., 2002). Here, aspects like peer relationships, peer acceptance, and pleasures derived from non-academic activities may be more relevant sources affecting emotions and well-being of adolescents opposed to specific learning outcomes such as academic achievement within the school context.

We further hypothesized that SWB predicts changes in students’ GPA. However, we did not find any predictive changes from any component of SWB on students’ academic achievement. The theoretical assumption that students are more academically successful the happier they are was not supported by our results. As suggested by other studies in this field, there are other factors aside from initial SWB that are more relevant for shaping adolescents’ academic functioning. Besides cognitive variables such as intelligence, several school-related variables such as different motivational and/or personality variables may be more relevant determinants of adolescents’ academic achievement (Steinmayr and Spinath, 2009). Moreover, students’ earlier and contemporaneous environments (Romero et al., 2014) might have a greater impact on the changes in academic achievement. For example, students from higher socioeconomic status (SES) homes have an advantage with regard to academic achievement outcomes over students from lower SES homes (Sirin, 2005), likely due to factors such as the value parents place on education (Eccles et al., 2004), the quality of teachers in public schools (Akiba et al., 2007), and neighborhood norms (Harding, 2003). Thus, these outside factors may yield stronger and more significant effects compared to the relationship between students’ well-being and school success.

Up until now, no study has investigated the reciprocal relationship between SWB and test anxiety. As outlined, we hypothesized that students’ test anxiety and SWB are reciprocally related. More precisely, we expected that worry and emotionality predict changes in the cognitive as well as the emotional component of SWB. This hypothesis could only be confirmed partly. Our results showed that only worry predicted changes in the cognitive as well as in the emotional components of SWB. This highlights the negative, maladaptive effects of worry on relevant life outcomes, such as SWB. Research has already demonstrated that especially worry has negative effects on academic achievement (e.g., Seipp, 1991; Eysenck et al., 2007). Our study is the first that demonstrated that negative thoughts, self-depreciating statements, and thinking about the negative consequences of failure regarding achievement situations are also detrimental for one’s SWB. However, emotionality, i.e., noticing physiological reactions according to testing situations, did not predict any changes in both components of SWB. Whereas emotionality may be a factor that mainly affects students when they are in an actual examination situation, worry may yield stronger effects students’ overall life circumstances and well-being. Thus, text anxiety interventions that focus on this test anxiety component (cf. von der Embse et al., 2013) might not only have a positive influence on academic achievement but also on students’ SWB.

We further hypothesized that SWB predicted changes in worry and emotionality. This hypothesis, however, could not be confirmed. Students who were dissatisfied with their life circumstances or display a rather negative mood did not necessarily develop symptoms of test anxiety. This also implies that a high level of SWB is no protective factor with regard to the development of test anxiety. Therefore, strategies for overcoming test anxiety should rather focus on reducing risk factors for developing test anxiety in school students, including ineffective coping strategies, poor self-beliefs, or other dispositional determinants, such as personality variables (e.g., Chamorro-Premuzic et al., 2008).

Finally, we examined the reciprocal relations between students’ test anxiety and academic achievement. We hypothesized that worry in particular predicts changes in students’ GPA. The results demonstrated that high levels of worry were associated with significantly lower achievement scores, and worry predicted negatively changes in students’ GPA. This is in line with major findings in test anxiety research demonstrating that the cognitive component of test anxiety is the factor most consistently found to be associated with declines in performance (Williams, 1991; Eysenck et al., 2007). This result can be explained by Eysenck’s Attentional Control Theory (Eysenck et al., 2007), which explains the negative effects of anxiety on students’ cognitive performance based on the idea that anxiety reduces students’ attentional focus to the examination task and instead makes a student focus his or her attention to other stimuli such as thoughts of worry.

Moreover, we hypothesized that students’ GPA predicts changes in worry and emotionality. This hypothesis, however, was not confirmed. Students’ GPA did not predict any changes in test anxiety. On the one hand, this implies that negative performances do not necessarily lead to a greater risk of becoming a test-anxious student. The idea that repeated difficulties with academic performances tend to lower students’ self-confidence, which in turn could create conditions for more frequent and intense experiences of test anxiety was not confirmed in our study. Although grades have become increasingly important in today’s society, determining students’ future career and job opportunities (e.g., Rana and Mahmood, 2010), grades may not be a predictive variable that determines the risk of becoming a test-anxious student. On the other hand, however, high achievement scores may not have a buffering effect for developing test anxiety. Although the causes of test anxiety in adolescence are not yet well understood, research shows that adolescents who are test-anxious tend to have high levels of general anxiety that are enforced during evaluative situations. For example, studies showed that some children and adolescents have biological predispositions to high levels of general anxiety making them more susceptible to the effects of being evaluated (Huberty, 2008). Thus, adolescents’ personality and biological predispositions may be more important in this relationship than school specific variables such as academic achievement.

In the full model we investigated the reciprocal effects between all constructs simultaneously and additionally controlled for gender. Effects did not change greatly when controlling for the other constructs. But most interestingly, gender predicted change in academic achievement and emotionality. Thus, on the one hand, our study contributes to the ongoing discussion on gender differences in scholastic achievement favoring girls (cf. Spinath et al., 2014). It seems that girls not only perform better in school in various phases in students’ academic careers observed in cross-sectional designs (Steinmayr and Spinath, 2009; Voyer and Voyer, 2014), but they are also better at improving their scholastic performance in one school track. At measurement occasion 2 students were 1.5 years away from graduating. Grades in this school phase already contribute to one’s GPA documented in the school leaving certificate in Germany which was not the case for the GPA at measurement occasion 1. Thus, girls seem to be able to improve their grades more successfully than boys in high stake phases. Future studies should investigate if this trend holds until students graduate from school. This process might contribute to their overrepresentation in selective studies as in Germany university selection mostly solely relies on a student’s school leaving certificate’s GPA. On the other hand, however, girls’ emotionality increased more strongly than boys’ emotionality scores. Thus, our study is the first that demonstrated that not only the association between gender and emotionality is stronger than between gender and worry (see also Zeidner, 1990) but that this also holds in a longitudinal approach and independent from the fact that girls have better grades than boys and are even able to increase this advantage. It might be that the fact that at measurement 2 school performance played a greater role for their future educational and vocational careers than ever and this high stake situation led to a stronger increase of emotionality in girls than in boys. Studies demonstrated that in high stakes situations children experience more test anxiety than in low stake situations (Segool et al., 2013). This impact of high stakes situation on test anxiety might be more pronounced for girls than for boys. This process might contribute to the gender gap favoring males in vocational success despite females’ greater academic success as emotionality is known to be a good negative predictor of vocational success (e.g., Aydin et al., 2005; Judge et al., 2013) and should be further investigated.

### Limitations and Conclusion

Although the findings are promising, there are several limitations to our study. First, the results should be replicated in other school tracks to ensure that they are generalizable. Our sample consists of students attending a Gymnasium, the highest school track in Germany. This sample is by no means representative of students in Germany because students from migration backgrounds and socially disadvantaged students are underrepresented at this kind of school. The sample is comparable to a university student sample. Thus, the present study is only a first step in understanding the importance of the reciprocal relationships between students’ well-being and the school variables test anxiety and academic achievement. Moreover, the present data is limited to the extent that a longitudinal study may not demonstrate a causal influence from one variable to another. The longitudinal design to which structural equation modeling is applied in the present study only allows for drawing conclusions about the pattern of change over time between the variables measured at each occasion in the investigated time lap. It does not represent a sufficient condition for the longitudinal impact of one variable on another because only experimental designs may reveal such definite causal effects. However, the finding of cross-lagged paths is yet a requirement for demonstrating the impact of one variable on another and is therefore important for planning any subsequent interventions (cf. Marsh et al., 1999). Moreover, another limitation to our study is the fact that we considered only two measurement occasions in the time span of 1 year so that no conclusions about changes within a school year or beyond can be made. Finally, we collected only self-report measures of the investigated variables of test anxiety and SWB. Future studies should collect additional measures of well-being, including teachers’ or parents’ reports on students’ emotional functioning.

Eventually, our findings highlight the need to further develop school-based interventions in the context of test anxiety. In the past, treatment programs for test-anxious students have generally involved helping students to deal with their emotional stress (Richard and Woolfolk, 1980). However, as we demonstrated the overall stronger impact of the worry component of test anxiety on students’ well-being and academic achievement, it is important to focus on interventions targeting cognitive factors. For example, recent studies demonstrated that especially the combination of relaxation and cognitive restructuring treatment may lead to the highest test performance in samples of students suffering from test anxiety (e.g., Akinsola and Nwajei, 2013). These findings indicated that cognitive restructuring techniques significantly reduced anxiety in a group of students with test anxiety and improved their post treatment test performance. Future studies in this field could not only try to design interventions that effectively reduce students’ test anxiety with positive effects on academic performance but also examine whether it may increase students’ well-being.

In sum, the present study tried to give insight into the background of the reciprocal relations between students’ SWB, academic achievement, and test anxiety. So far, no single longitudinal study examined the reciprocal relationship between students’ test anxiety and SWB over time, and only a few studies on the association between academic achievement and SWB have been carried out before. However, the study design still leaves open questions about whether the investigated variables actually cause effects on each other. Future research should try to manipulate the investigated variables to further explore the causal relationships between test anxiety, academic achievement, and SWB.

Finally, our study demonstrated that test anxiety, in particular worry, as well as students’ GPA have powerful predictive qualities with regard to changes in students’ SWB, which reflects the need for further research in the context of education intervention programs and strategies to treat test anxiety successfully and thereby positively influence adolescents’ well-being.

## Conflict of Interest Statement

The authors declare that the research was conducted in the absence of any commercial or financial relationships that could be construed as a potential conflict of interest.
